# CD32-RNA Co-localizes with HIV-RNA in CD3+ Cells Found within Gut Tissues from Viremic and ART-Suppressed Individuals

**DOI:** 10.20411/pai.v4i1.271

**Published:** 2019-05-03

**Authors:** Joshua J. Vásquez, Brandon L. Aguilar-Rodriguez, Leonardo Rodriguez, Louise E. Hogan, Ma Somsouk, Joseph M. McCune, Steven G. Deeks, Zoltan G. Laszik, Peter W. Hunt, Timothy J. Henrich

**Affiliations:** 1 Department of Medicine, Division of Experimental Medicine, University of California, San Francisco; 2 Department of Medicine, Division of Pulmonary, Critical Care, Allergy, and Sleep Medicine, University of California, San Francisco; 3 Department of Medicine, Department of Medicine, Division of HIV, Infectious Diseases, and Global Medicine, University of California, San Francisco; 4 Department of Medicine, Division of Gastroenterology, University of California, San Francisco; 5 Department of Pathology, University of California, San Francisco; * These two authors contributed equally to this work

**Keywords:** HIV, CD32, gut tissue, HIV reservoirs, HIV-RNA, in situ hybridization

## Abstract

**Background:**

Identifying biomarkers for cells harboring replication-competent HIV is a major research priority. Recently, there have been mixed reports addressing the possibility that CD32-expressing T cells are enriched for HIV. There is growing evidence that CD32 expression increases with cellular activation that may be related to, but not necessarily specific for, infection with HIV. However, the relationship of CD32 expression to HIV-infection in subtypes of tissue-resident leukocytes is unclear.

**Methods:**

First, we used duplex chromogenic in situ hybridization to identify cells actively transcribing RNA for both CD32 and HIV on human gut tissues. Then we performed multiplexed immunofluorescence and in situ hybridization (mIFISH) on sections from the same tissues to determine the phenotype of individual cells co-expressing HIV-RNA and CD32-RNA.

**Results:**

HIV-RNA+ cells were more abundant in tissues from viremic individuals than in those receiving suppressive anti-retroviral therapy (ART). However, staining by both methods indicated that a higher proportion of HIV-RNA+ cells co-expressed CD32-RNA in ART-suppressed individuals than in those with viremia. The majority of HIV-RNA+ cells were CD3+.

**Conclusions:**

Our data suggest that the transcription of CD32-RNA is correlated with HIV transcriptional activity in CD3+ cells found within human gut tissue. Whether or not up-regulation of CD32-RNA is a direct result of HIV transcription or more global T-cell activation remains unclear.

## INTRODUCTION

Whereas cell populations harboring HIV in the peripheral blood have been studied extensively [1], tissue reservoirs are less well described and are thought to exist within tissue resident lymphocyte subsets that may not be found in circulating blood [2, 3]. Identification and characterization of cell-surface biomarkers of HIV-infected cells are major research priorities that will enable development of strategies to target and eliminate HIV reservoirs [4]. CD32a has been described as a potential biomarker for circulating HIV-infected CD4+ T cells [5]. CD32a gene transcription was initially observed to be highly upregulated in HIV-infected cells in an *in vitro* primary CD4+ T-cell HIV latency model, and CD4+ T cells from HIV-infected individuals receiving antiretroviral therapy (ART) were immunostained and sorted based on cell surface expression of CD32 (not specific for the CD32a isoform) and were highly enriched in HIV-DNA [5]. However, recent observations suggest that the expression of CD32 on T cells may be associated with activation that is not specific to HIV infection [6]. Moreover, other reports question the possibility that T cells expressing CD32 are enriched for HIV [7-10]. However, technical differences in cellular pheno-typing, isolation, and measures of the reservoir make comparisons across these works difficult to interpret [7, 9, 11]. Also, 1 group showed that RNA for CD32 and HIV co-localize in human lymph nodes [12]. However, since CD32 is most frequently associated with myeloid cells, the questions remain regarding the phenotype of CD32+ HIV-infected cells and the extent to which CD32 co-localizes with HIV-infected cells in other tissues. To explore these issues further we sought to assess the transcriptional activity of CD32-RNA and HIV-RNA in gut tissue from viremic and ART-suppressed HIV-infected individuals using RNAscope in situ hybridization (ISH) or multiplexed immunofluorescence with ISH (mIFISH) [13].

## METHODS

### Study Population and Gut biopsies

Gut tissue was obtained through the UCSF SCOPE cohort by colonoscopy in which 3-mm sections were taken from the rectum and/or ileum using jumbo forceps. Participants ≥ 18 years of age were enrolled and clinical data obtained included history of ART, viral load measurements, and CD4+ T-cell counts, race/ethnicity, age, and detailed medical histories. Tissue used in this study included rectal and/or ileal biopsies from HIV-infected participants receiving ART (n = 4, including rectal from 4 and ileal from 3) and from those who were not (n = 2 rectal), one aviremic HIV controller (rectal), and one HIV negative control (rectal). Tissue was promptly preserved in 4% paraformaldehyde before paraffin embedding and sectioning for ISH and mIFISH, as previously described [13].

### Ethics Statement

The UCSF SCOPE cohort provided a source of gut biopsies and was approved by the UCSF Committee for Human Research. All volunteers provided written informed consent.

### In Situ Hybridization of Ileal and Rectal Tissue

Sections from formalin-fixed, paraffin-embedded (FFPE) gut tissues were stained using RNA-scope branched-DNA ISH technology distributed by Advanced cell Diagnostics (ACD), Newark, CA. The RNAscope assay was performed on 2- or 4-µm-thick sections using the RNAscope 2.5 HD single-plex red kit, the 2.5 duplex red-black kit, or a custom-duplex fluorescent kit as previously described [13]. RNAscope probes targeting HIV-RNA (ACD Catalogue No. 416111) and the anti-sense of the CD32 gene (ACD Catalogue No. 555921) were developed by ACD and used according to the manufacturer's recommendations. For mIFISH assays primary antibodies against human CD3, CD68, and CD163 as well as appropriate secondary antibodies were used as previously described [13]. Bright-field whole-slide digital images of stained sections were obtained using the Leica Aperio slide scanner while fluorescent images were obtained using the Zeiss Axioscan Z.1 slide scanner. The total number of HIV-RNA^+^ or HIV-RNA^+^/CD32-RNA^+^ cells was manually counted and phenotyped where possible. The total number of hematoxylin or DAPI+ nuclei in each section was assessed using the Definiens Tissue Studio software (Munich, Germany).

### Statistical Methods

Since we had both ileal and rectal tissues from some of the participants (ie, data clustered by participant), we used linear mixed models to compare each analyte between groups, transforming outcome variables as necessary to satisfy model assumptions. This approach also allowed us to simultaneously adjust for anatomical location (ie, ileum vs rectum). Analyses were performed using Stata SE, version 15, College Station, TX.

## RESULTS

**Figure 1.**
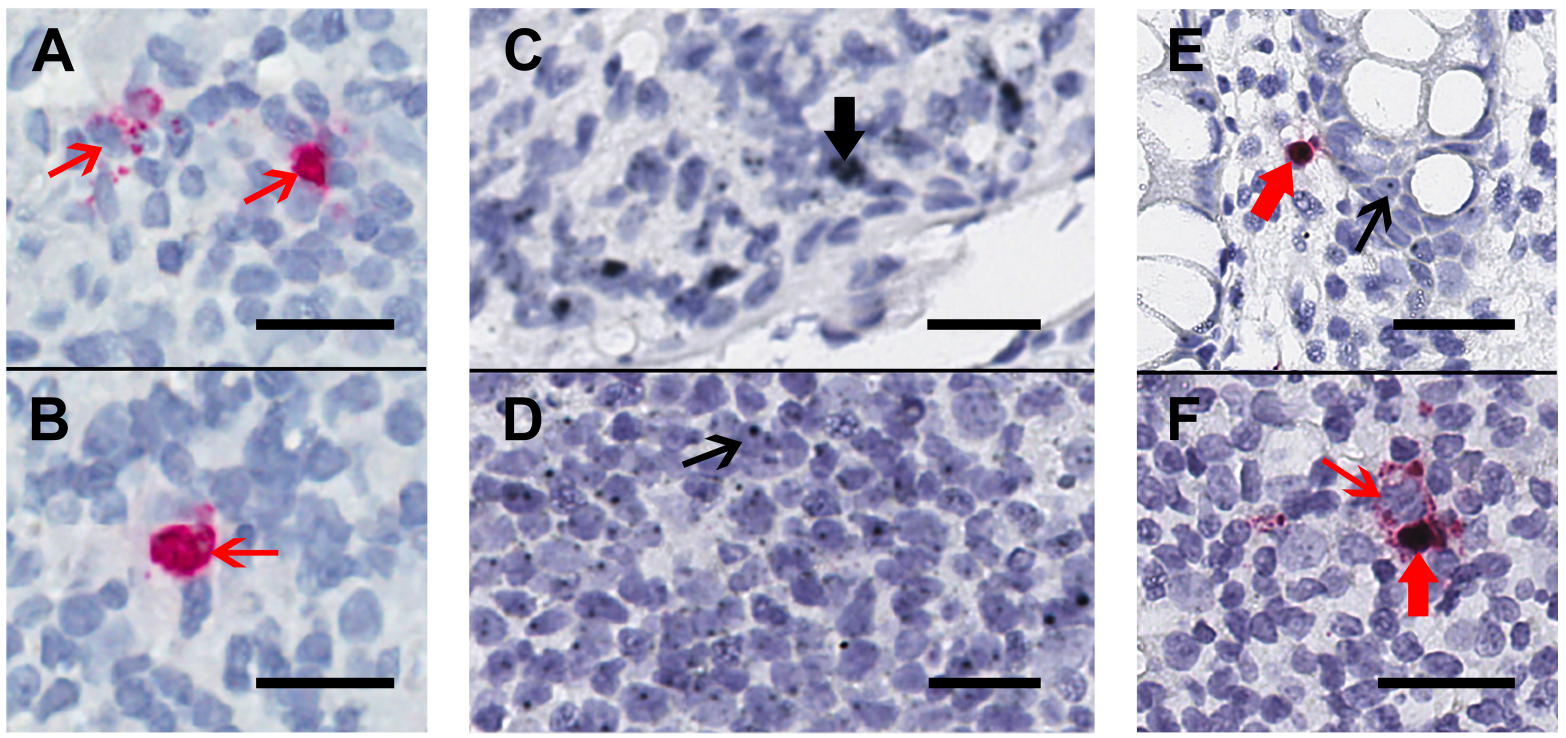
**Chromogenic ISH staining for HIV-RNA alone or in duplex with CD32-RNA and HIV-RNA on human gut tissue.** Human gut tissue was stained for HIV-RNA (red) alone (Panels A and B) or CD32-RNA (black) and HIV-RNA (red) (Panels C-F), with nuclei (blue). Panels A and B, a rectal biopsy from an HIV-infected viremic donor that was stained for HIV-RNA alone demonstrates 2 separate areas of a single slide showing high transcription of HIV-RNA (thin red arrows). Staining with the red-chromogen alone was used to distinguish signals from the co-localization of the black and red chromogens. Panel C and D, a rectal biopsy from an HIV-negative donor showing regions containing cells with high CD32-RNA signal (panel C, thick black arrow) and low CD32-RNA signal (panel D, thin black arrow). Panel E, a rectal biopsy from an HIV-positive ART-suppressed donor shows an HIV-RNA positive cell that is also positive for CD32-RNA (thick red arrow), and nearby HIV-RNA negative cells show low CD32-RNA signals (thin black arrow). Panel F, a rectal biopsy from an HIV-positive viremic donor shows adjacent HIV-RNA positive cells that are either highly positive for CD32-RNA staining (thick red arrow) or negative for CD32 staining (thin red arrow). Scale bars = 20 μm

**Figure 2.**
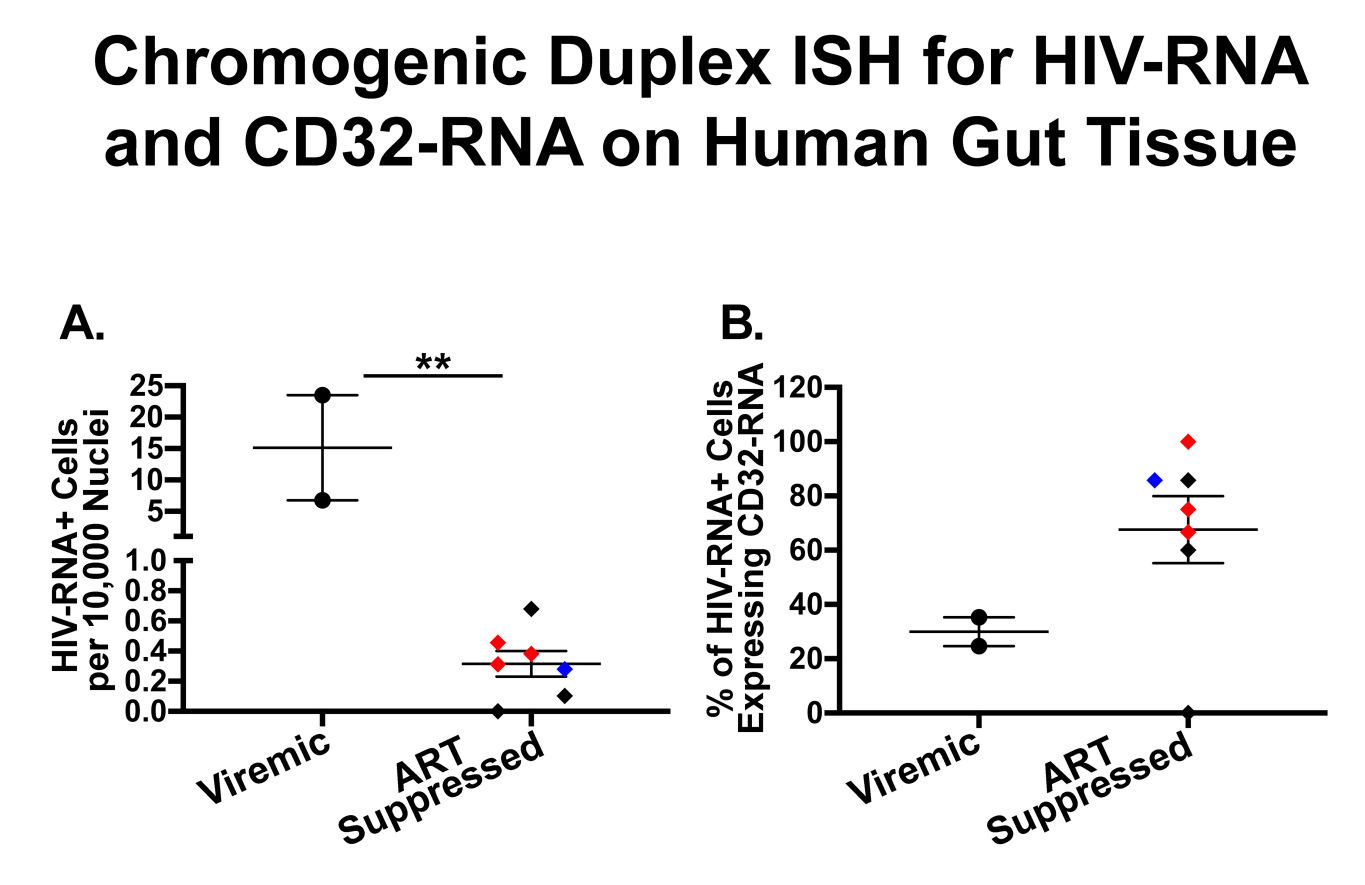
**Quantification of cells expressing both HIV-RNA and CD32-RNA in human gut tissues stained by duplex chromogenic ISH.** Human gut tissues from viremic (circles) and ART-suppressed (diamonds) individuals were stained for HIV-RNA and CD32-RNA by duplex chromogenic ISH. In this figure each tissue is represented by an individual symbol where black = rectal tissues, red = ileal tissues, and the blue diamond = rectal tissue from an elite controller. The error bars show the standard error of the mean. Panel A, The number of HIV-RNA+ cells per 10,000 nuclei was much higher in viremic compared to ART-suppressed tissues [median: 15.1 (range: 6.8-23.5) vs 0.31 (range: 0-0.7), respectively] (*P* < 0.001). Panel B, The percentage of total HIV-RNA+ cells expressing CD32-RNA tended to be higher in tissues from ART-suppressed donors compared to viremic tissues [median: 75% (range 0%-85%) vs 30% (range 25%-35%), respectively] (*P* = 0.079). There was no significant difference in the number of HIV-RNA+ cells per 10,000 nuclei (*P* = 0.45) or the percentage of HIV-RNA+ cells expressing CD32-RNA (*P* = 0.58) in ileal vs rectal tissues. Tissues from HIV negativeindividuals did not show staining for HIV-RNA.

We collected rectal and/or ileal biopsies that were formalin-fixed, paraffin-embedded from HIV-infected participants receiving ART (n = 4, including rectal from 4 and ileal from 3) and those who were not (n=2 rectal), one aviremic HIV controller (rectal), and one HIV negative control (rectal). First, these tissues were stained using chromogenic RNAscope ISH staining for HIV-RNA alone (Figure 1A) or by duplex-ISH for CD32-RNA and HIV-RNA on 4-µm-thick sections (Figures 1B-D). Single stains of HIV-RNA were used as a control to ensure that expression of HIV-RNA alone (Figure 1A-B) could be distinguished from either CD32-RNA alone (Figures 1C-D) or co-localization of HIV-RNA and CD32-RNA (Figures 1E-F). Overall, CD32-RNA staining in HIV-RNA negative cells across tissues was highly variable (both anatomically and by sample) and did not appear to correlate with the frequency of HIV-RNA positive cells. In addition, CD32-RNA staining in individual cells was also variable and included cells with signal throughout (eg, Figure 1C) as well as small punctate signals within the nucleus (eg, Figure 1D). Interestingly, HIV-RNA+ cells were among the cells with the strongest CD32-RNA signal and had significant extra-nuclear CD32 signal foci. ART-suppressed participants had a mean 93% lower frequency of HIV+ cells per 10,000 nuclei than viremic individuals (P < 0.001), without evidence for a difference between ileal and rectal sites (P = 0.45) (Figure 2A).

Although quite variable, a substantial fraction of HIV-RNA+ cells co-expressed CD32-RNA in both ART-suppressed and viremic tissues, and perhaps more so in ART-suppressed individuals (Figure 2B). However, that difference was not statistically significant (*P* = 0.079). Also, there was no evidence for a difference between ileal and rectal sites (*P* = 0.58).

**Figure 3.**
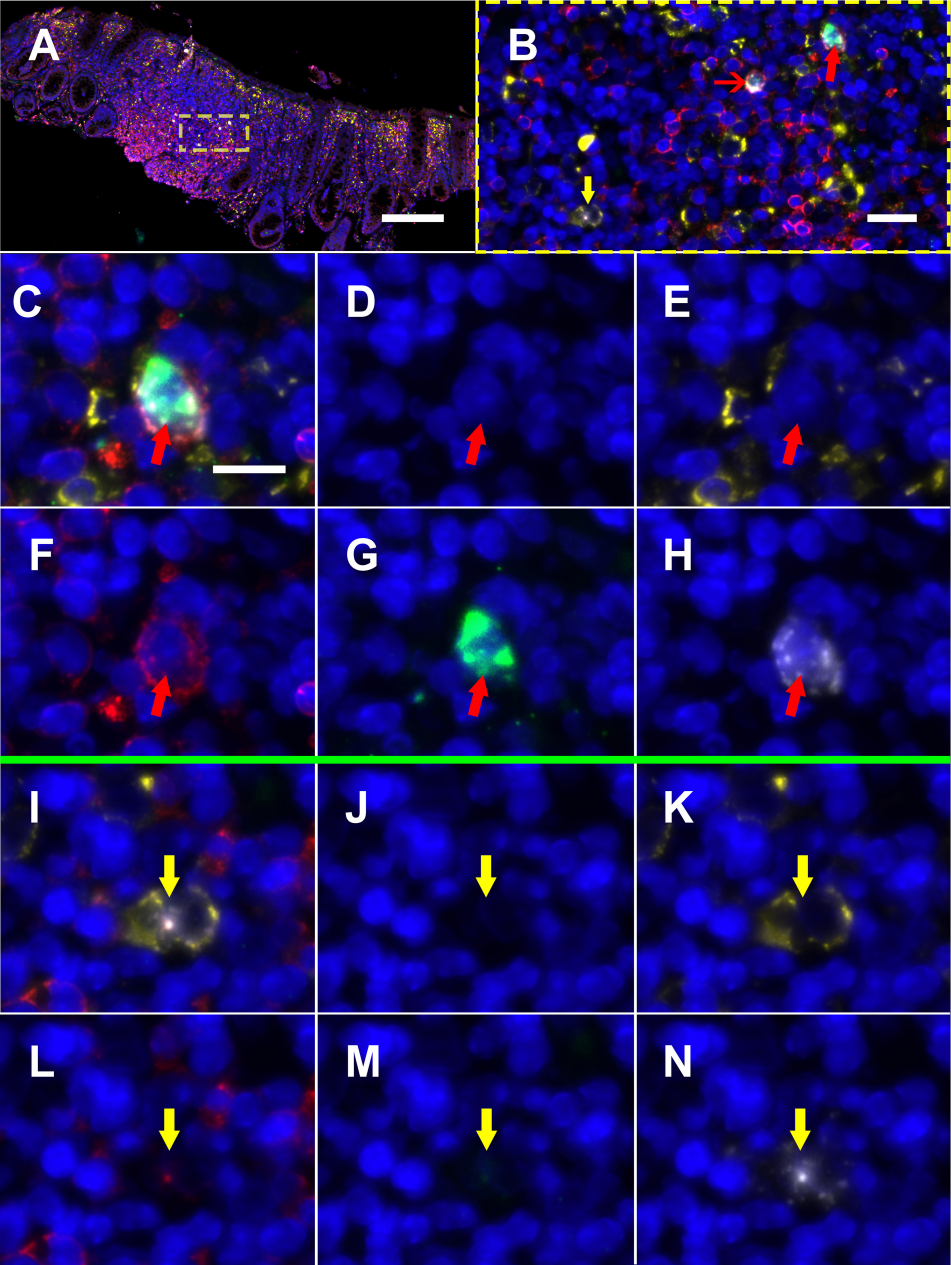
**Multiplexed immunofluorescence and in situ hybridization (mIFISH) staining for HIVRNA, CD32-RNA, CD3, and CD68/CD163 on viremic human gut tissue.** HIV-RNA (white), CD32-RNA (green), CD3 (red), CD68/CD163 (yellow), with nuclei (blue). Panel A, a low-power overlay of mI-FISH-stained human gut tissue from an HIV+ viremic donor. Panel B, a higher-power image of the ROI in the yellow square showing 2 HIV-RNA+ cells, CD3+ cells that stained for both HIV-RNA and CD32-RNA (red arrows), and a CD68/CD163+ cell that stained for HIV-RNA alone (yellow arrow). The cells with the thick red arrow and yellow arrow are then shown individually at high-power by channel (overlay, DAPI, CD163/38, CD3, CD32-RNA, and HIV-RNA). Panels C-H, a CD3+/HIV-RNA+/CD32-RNA+ cell (thick red arrow). Panels I-N, a CD68/CD163+/HIV-RNA+ cell (yellow arrow). Scale bar for panel A = 200 μm; scale bar for panel B = 20 μm; scale bar for panels C-N = 10 μm.

**Figure 4.**
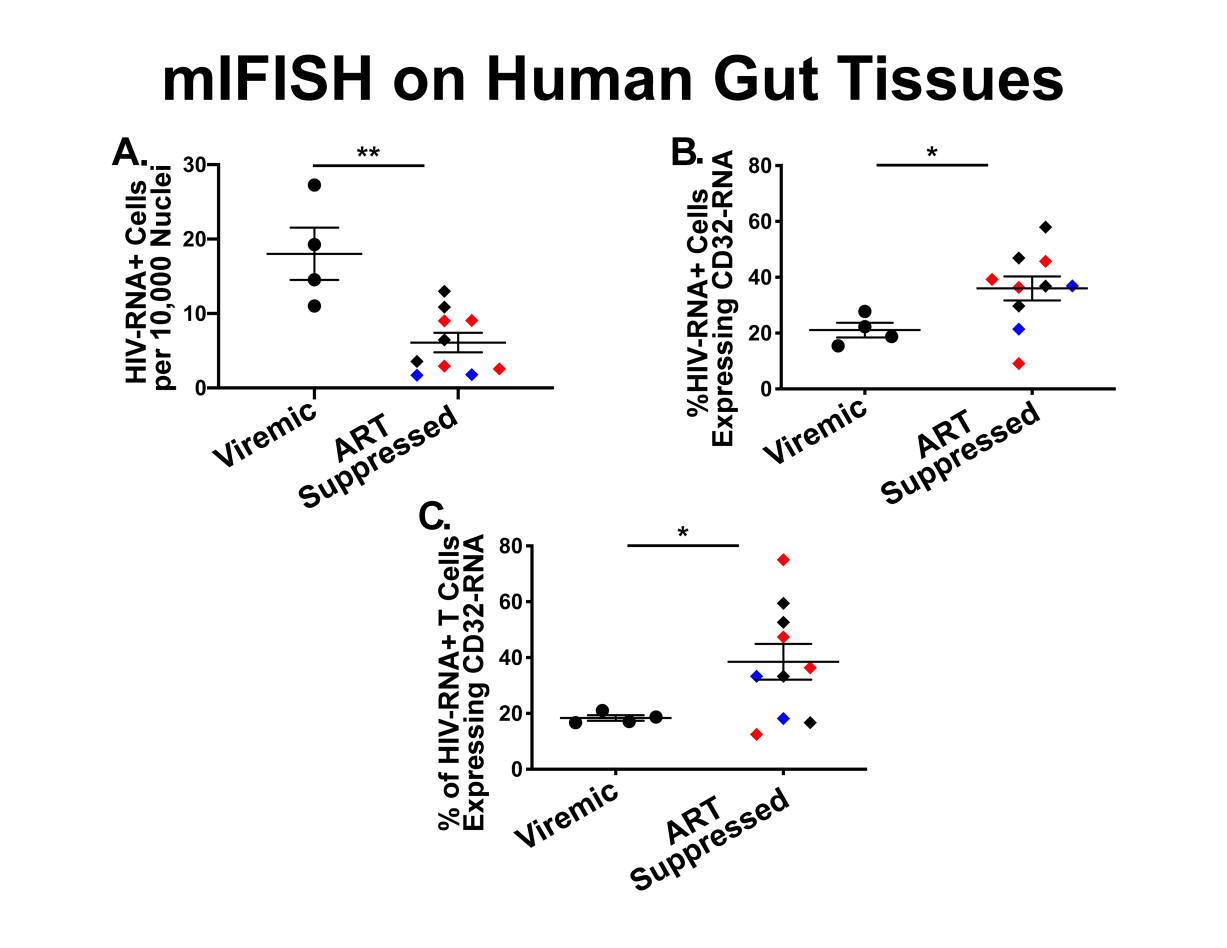
**Quantification of HIV-RNA positive cells with and without transcription of CD32-RNA stained using multiplexed immunofluorescence and in situ hybridization (mIFISH).** Human gut tissues from viremic (circles) and ART-suppressed (diamonds) individuals were stained by mIFISH for HIVRNA, CD32-RNA, CD3, and CD68/CD163. In this figure each tissue section is represented by an individual symbol where black = rectal tissues, red = ileal tissues, and the blue diamonds = rectal tissue from an elite controller. The error bars show the standard error of the mean. Total HIV-RNA+ cells were increased in viremic compared to ART-suppressed individuals [median: 17 (range 11-27) vs 5 (range 2-13) cells/10,000 nuclei, respectively] (*P* = 0.0017)] (Panel A). Both the percentage of total HIV-RNA positive cells (Panel B) and of HIV-RNA+/CD3+ cells (Panel C) expressing CD32-RNA was significantly higher in tissues from ART-suppressed donors when compared to viremic donors [median % total HIV-RNA+ cells: 37% (range 9%-60%) vs 21% (range 15%-28%), respectively, *P* = 0.0122; and [median % CD3+/HIV-RNA+ cells: 35% (range 13%-75%) vs 18% (range 17%-21%), respectively, *P* = 0.0119]. Tissues from HIV negative individuals did not show staining for HIV-RNA.

**Figure 5.**
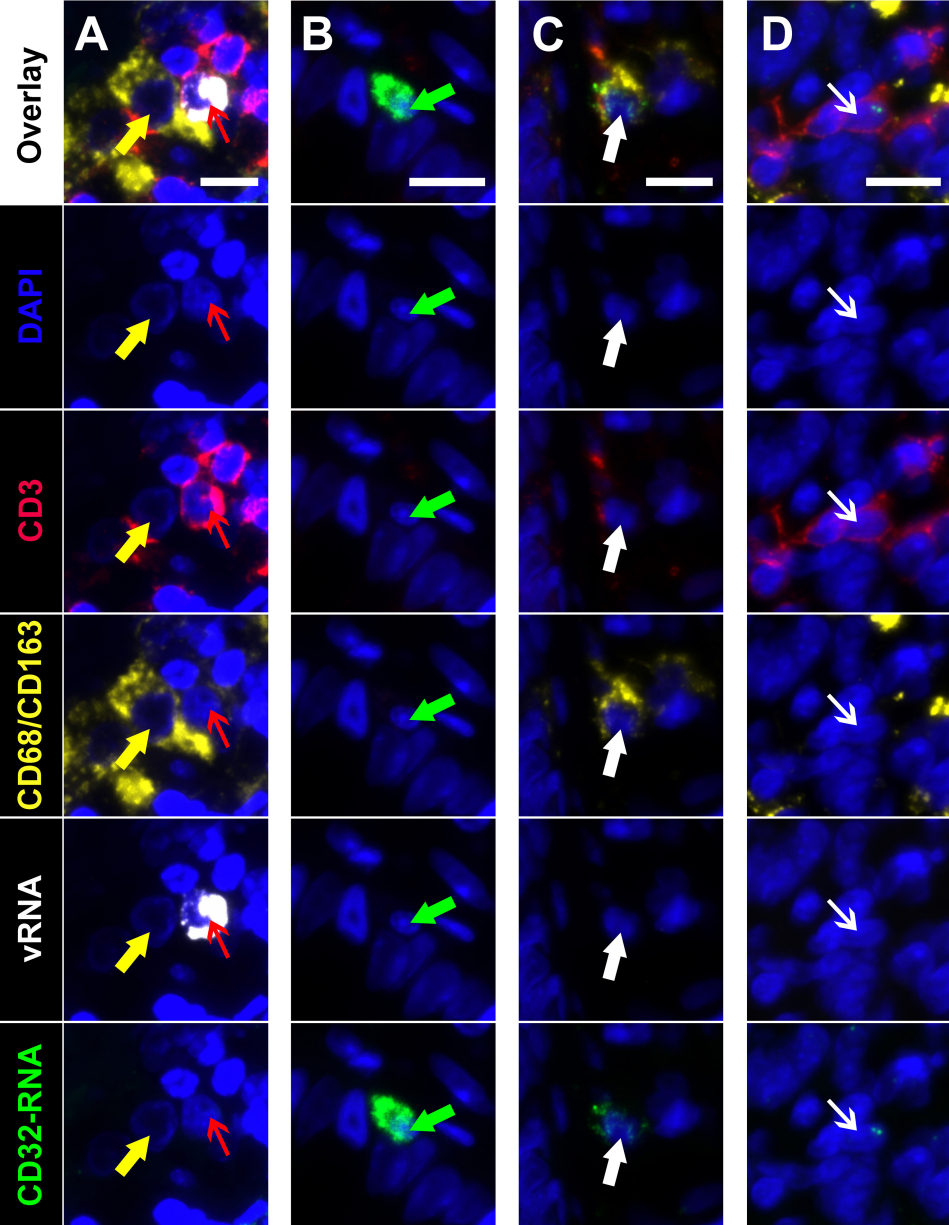
**Multiplexed immunofluorescence and in situ hybridization (mIFISH) identifying cells of interest in gut tissue from an HIV+ viremic individual. Individual regions of interest are arranged in columns with the channels visualized identified by row.** HIV-RNA (white), CD32-RNA (green), CD3 (red), CD68/CD163 (yellow), with nuclei (blue). Column A, an HIV-RNA-/CD32-RNA-macrophage (yellow arrow) that appears to be phagocytosing an HIV-RNA+/CD32-RNA-T cell (red arrow). Column B, an unphenotyped cell that is highly positive for CD32-RNA signal but negative for all other markers (green arrow). Column C, a macrophage with low level positivity for CD32-RNA (thick white arrow). Column D, a CD3+ T cell that has low level positivity for CD32-RNA but is negative for all other markers (thin white arrow). Scale bars = 10 μm.

Next, to determine the phenotype of cells co-expressing RNA for both HIV and CD32 we performed mIFISH using previously established protocols [13]. In short, duplex fluorescent ISH for HIV-RNA and CD32 RNA was followed by immunofluorescent (IF) staining (Figure 3) for CD3+ T cells (Figures 3D-I) and CD68/CD163+ macrophages (Figures 3J-O) in batch on 2 sections 2 µm thick from all but 1 of the individuals described above. After whole-slide digital imaging, we manually quantified the number of cells expressing HIV-RNA or co-expressing RNA for HIV and CD32 and phenotyped each cell when possible. Nuclei were quantified using the Definiens Tissue Studio software. HIV-RNA+ cells were found at a median frequency of 17 (range 11-27) and 5 (range 2-13) cells/10,000 nuclei in tissues from viremic vs ART-suppressed participants, respectively (*P* = 0.008) (Figure 4A). There was no difference in the frequency of HIV-RNA+ cells between ileal and rectal tissues from ART-suppressed individuals. Among viremic participants, HIV-RNA staining was concentrated in lymphoid aggregates with nearby clusters of HIV-RNA+ cells, while among ART-suppressed participants, HIV-RNA+ cells tended to be found in isolation and outside follicular structures. A higher mean percentage of HIV-RNA+ cells expressed CD32-RNA in ART-suppressed than in viemic individuals (36% vs 21%, *P* = 0.025) (Figure 4B). There was no evidence for an effect of ileal vs rectal origin on the proportion of HIV-RNA+ cells that expressed CD32 in ART-suppressed individuals. Approximately 70% of the 1,399 HIV-RNA+ cells identified across all of the samples could be phenotypically identified, of which the vast majority were CD3+ T cells. The percentage of HIV-RNA+ cells expressing CD32-RNA continued to be higher in ART-suppressed than in viemic individuals, even when restricting to CD3+ T cells *(P =* 0.033) (Figure 4C), but this difference was attenuated when adjusted for the gastrointestinal region *(P =* 0.12). Just 13 HIV-RNA+ cells were identified as CD68/CD163+ macrophages (0.18 and 0.02 cells per 10,000 nuclei in samples from viremic and ART-suppressed participants, respectively), and only 2 of those cells were CD32-RNA+. We cannot be certain that macrophages associated with HIV-RNA did not also harbor phagocytosed T cells. However, we were able to distinguish HIV+ T cells from HIV-macrophages in close proximity. Figure 5A, by example, depicts an HIV+ T cell that appears to be undergoing phagocytosis from a nearby HIV-macrophage. The subpopulation of CD32-RNA+/HIV-RNA-cells was too numerous to count manually, but it was visually evident that the overwhelming majority of these cells were negative for both CD3 and CD68/CD163 IF markers (Figure 5B). Finally, while the total number of cells expressing CD68/CD163 or CD3 were not quantified, careful visual inspection revealed that only a minority of the total CD68/CD163+ cells expressed CD32-RNA (Figure 5C), and CD3+/HIV-RNA-cells expressing CD32-RNA were exceedingly rare (Figure 5D). Moreover, the signal intensity for CD32-RNA observed in CD3+/HIV-RNA-cells (Figure 5D) was overall much lower than in CD3+/HIV-RNA+ cells (Figure 3D-I). A nuclear pattern of CD32-RNA staining was observed by fluorescent staining similar to the chromogenic staining (Figure 1D) and included cells with lower levels of CD32-RNA signal such as the CD3+/HIV-RNA-cells (Figure 5D).

## DISCUSSION

These data reveal co-localization of HIV and CD32 transcriptional activity in tissue-resident cells from HIV-infected individuals and are in line with recent studies performed in the lymph node [12]. Extending those studies to another proposed anatomic reservoir of HIV, the gut, we describe a higher proportion of HIV+ transcriptionally active cells co-expressing CD32-RNA from ART-suppressed compared to viremic individuals. Importantly, our findings suggest that co-localization of RNA for CD32 and HIV is less likely explained by myeloid-cell-associated HIV (eg, via direct infection or phagocytosis of infected cells) as nearly all of the cells co-expressing RNA for HIV and CD32 that could be phenotypically identified were CD3+. This is in contrast to studies performed in PBMCs by flow cytometery that suggest that HIV+/CD32+ cells may actually represent doublets associated with CD32+ B cells [8]. We found that the vast majority of HIV-RNA-/ CD32-RNA+ cells were negative for both T-cell and macrophage markers and thus may have been B cell in origin. Notably, we observed that CD3+ cells that were HIV-RNA-/CD32-RNA+ were rare and were among the cells with the lowest CD32 transcriptional activity. CD32-RNA+ staining frequently appeared as a single nuclear signal and may indicate occasional binding of the probe to a complementary DNA target or potentially off-target hybridization, although the RNAscope probe design and our protocols that include several background reducing considerations (eg, binding temperatures, salt concentration) should minimize both of these issues [13]. However, it is also possible that this pattern reflects post-transcriptional regulation of CD32-RNA (eg, rapid degradation or decreased nuclear export) and is less likely to occur in HIV-RNA+ cells or other cells with increased CD32 transcriptional activity. Other groups have demonstrated that the expression and degradation of CD32-mRNA isotypes and protein subunits is highly-regulated and differs across various cell types and activation states [14-19], including CD32a-mRNA in activated T cells [20]. Moreover, similar to previous observations in the lymph node [12] we found that cells co-expressing RNA for CD32 and HIV were among the cells with the most intense staining for CD32-RNA. Thus, the significant proportion of CD3+ cells co-expressing RNA for both HIV and CD32, and the intensity of the CD32 staining, indicate a link between active transcription of HIV and the CD32 gene. Whether or not up-regulation of CD32-RNA is a direct result of HIV transcription or more global T-cell activation is not known. Taken together these findings are consistent with previous reports that the majority of HIV-infected cells in tissues are CD4+ T cells [13, 21, 22], and that in uninfected individuals, surface protein expression of CD32 on resting T cells is rare (~1%) and primarily seen following cellular activation [20, 23, 24].

This study has important limitations. First, the CD32-RNA ISH probes recognize all 3 CD32 gene products (ie, FcγRIIA, FcγRIIB, and FcγRIIC) and cannot distinguish differential transcriptional activity of the CD32a gene, which itself has been associated with HIV infection [5, 12]. Unfortunately, IF staining for CD32 by mIFISH was unsuccessful, as the compatibility of IF markers with mIFISH depends on the properties of the antibodies and their targets. Therefore, the findings of this transcriptionally-based study do not take into account downstream translational events or post-translational modifications. Additionally, it is possible that co-expression of CD32 and HIV RNA may be independent events that identify a sub-population of activated cells, or represent a sub-population of CD32+ cells that were at higher risk of infection with HIV. However, an increased frequency of HIV-/CD32+ T cells may be expected if HIV-unrelated events lead to increased CD32 transcriptional activity. Furthermore, the number of HIV-RNA+ cells was lower in mIFISH than in chromogenic staining, an observation that may be due to technical differences in sensitivity or because of the thinner sections stained by mIFISH (2 vs 4 µm). Also, it should be noted that this study includes a relatively small sample size that limits statistical power. However, since each staining protocol was performed and imaged in batch we were able to limit run-to-run variability. Finally, just 70% of the total HIV-RNA positive cells could be positively identified by IF which may be due to technique and/or a decrease or absence of protein expression for the relevant markers in infected cells. Nonetheless, our data suggest that the presence of CD32-RNA is correlated with HIV transcriptional activity in CD3+ cells found within human gut tissues.
